# Synergistic Effect of Polyethylene Glycol and Lactic Acid on Handling Properties and Antibacterial Efficacy of Premixed Calcium Silicate Cement

**DOI:** 10.3390/jfb15070187

**Published:** 2024-07-05

**Authors:** Yi-Huei Huang, I-Ting Wu, Chun-Cheng Chen, Shinn-Jyh Ding

**Affiliations:** 1 School of Dentistry, Chung Shan Medical University, Taichung City 402, Taiwan; yihui51580@gmail.com; 2 School of Dentistry, China Medical University, Taichung City 404, Taiwan; 3 Department of Dentistry, China Medical University and Hospital, Taichung City 404, Taiwan; 4 Department of Stomatology, Chung Shan Medical University Hospital, Taichung City 402, Taiwan; 5 Institute of Oral Science, Chung Shan Medical University, Taichung City 402, Taiwan

**Keywords:** premixed cements, calcium silicate, rapid setting, anti-washout, antibacterial activity

## Abstract

Calcium silicate (CaSi) bone cement with antibacterial and osteogenic properties has attracted significant interest. However, there is a need to develop a variety of new premixed bone cement to meet the clinical requirements of fast setting time, ease of handling, and efficient antibacterial properties. In this study, different volume ratios of polyethylene glycol (PEG) and lactic acid liquids were added to calcium silicate, and the effects of varying liquid-to-powder ratios (L/P) were examined. This study assessed the physicochemical properties, cytotoxicity, and antibacterial activity against *S. aureus* and *E. coli* of this premixed cement. The results from the experiments indicated that lactic acid significantly reduced the setting time of the CaSi-based cement and enhanced its mechanical strength. Furthermore, the appropriate concentration of lactic acid and matching L/P ratio improved its washout resistance. The cell viability of all premixed cement was found to be over 80%. The premixed cement containing PEG and lactic acid exhibited superior antibacterial properties compared to the CaSi control. Based on its setting time, washout resistance, and antibacterial activity, a premixed cement with a liquid phase of 80% PEG and 20% lactic acid at an L/P ratio of 0.4 appeared promising for use in dental and orthopedic practice.

## 1. Introduction

Conventional bone cement is a reliable biomaterial for treating bone diseases like trauma or osteoporosis. It helps support weakened bones and acts as a scaffold for regenerative cells to attach to. The most common type of bone cement is calcium phosphate bone cement, which has good strength and osteoconductivity [1,2]. After setting, calcium phosphate forms hydroxyapatite, the primary component of bone. Although this traditional bone cement has many benefits, it also has some limitations that need to be addressed. Firstly, traditional bone cement requires on-site water–powder mixing. Subsequently, it needs to be transferred into the bone defect before hardening outside. This may lead to inhomogeneous water–powder mixing and prolong the surgical time [1,3]. Secondly, on-site water–powder mixing demands aseptic instruments and environment, making the surgical operation more complicated and time-consuming. To conquer these drawbacks, the first premixed calcium phosphate bone cement was developed in 2003 [1].

Premixed (ready-to-use) bone cement is a type of cement whose powder is mixed with a non-aqueous (water-free) liquid to form a premixed paste [1,3]. The paste only starts to harden when exposed to an aqueous environment [4], ensuring long-time storage in proper packaging. After this paste is transferred into the human body, it absorbs water from body fluid and hardens gradually to become cement type. Thus, there is no need to manually mix the powder with water during surgical operations. The water-free liquid creates space between powder particles, allowing water to penetrate and replace the water-free liquid [4]. The water-free liquid can be either water-miscible [3,5,6] or water-immiscible [4,7]. Polyethylene glycol (PEG) and glycerol typically serve as the water-miscible liquid of premixed cement due to their non-toxic and biocompatible properties. It is worth noting that PEG is commonly used in drug manufacturing and possesses an anti-fouling ability [8].

Calcium silicate-based cement, unlike calcium phosphate, exhibits a promising bacteriostatic capability by alkalizing the environment through the production of calcium hydroxide when it undergoes hydration reaction [9,10,11,12,13]. It is also known to induce stem cells to grow, proliferate, and differentiate more effectively than other materials [10,14,15]. Furthermore, calcium silicate (CaSi) has a remarkable remineralization ability and can stimulate bone regeneration [16,17,18,19,20,21,22]. In 2011, Persson et al. conducted the first examination of premixed CaSi for endodontics [23]. Later, Zhou et al. [24] and Wu et al. [25] improved the properties of premixed CaSi cement containing phosphates, such as setting time and anti-washout ability. They also added zirconia to make it radiopaque, which fulfilled the clinical requirements of root canal filling material. On the other hand, a setting accelerator is commonly used to reduce setting time. Xu and his colleagues conducted a study on premixed calcium phosphate cement that contained different acids [3]. The use of acids such as citric acid, tartaric acid, and malonic acid significantly accelerated the setting time and improved the strength of the cement. Other studies have shown that lactic acid can accelerate the setting reaction of CaSi [26,27]. Importantly, lactic acid is a compound in human metabolism and has been approved by the FDA for use in pharmaceuticals and cosmetics [28,29].

Our study, conducted with attention to detail, aimed to explore the synergistic effect of PEG and lactic acid on the physicochemical and biological properties of premixed CaSi cement. To this end, we introduced different concentrations of lactic acid in non-aqueous liquid (PEG) to the premixed CaSi cement at different liquid–powder ratios (L/P). We assessed the phase composition, microstructure, setting time, mechanical property, pH value, and anti-washout ability. Murine L929 fibroblast cells were used to evaluate the cytotoxicity of the cement. At the same time, antibacterial tests were conducted against Gram-positive *Staphylococcus aureus* (*S. aureus*) and Gram-negative *Escherichia coli* (*E. coli*) to evaluate the bacteriostasis of the cement. 

## 2. Materials and Methods

### 2.1. Preparation of Casi Powder

The study used CaSi powder containing 10% carboxymethyl cellulose (Wako, Osaka, Japan) to make premixed cement. The CaSi powder was synthesized using the sol-gel method described in a previous study [30]. To make the powder, nitric acid, reagent grade tetraethyl orthosilicate, absolute ethanol, and Ca(NO_3_)_2_ were mixed and incubated at 60 °C for one day, followed by drying in a 120 °C oven. The dried solids were sintered at 800 °C, milled in absolute ethanol for 12 h, and the ethanol was evaporated at 60 °C to obtain the CaSi powder. 

### 2.2. Preparation of Premixed Paste and Cement

**Figure 1:**
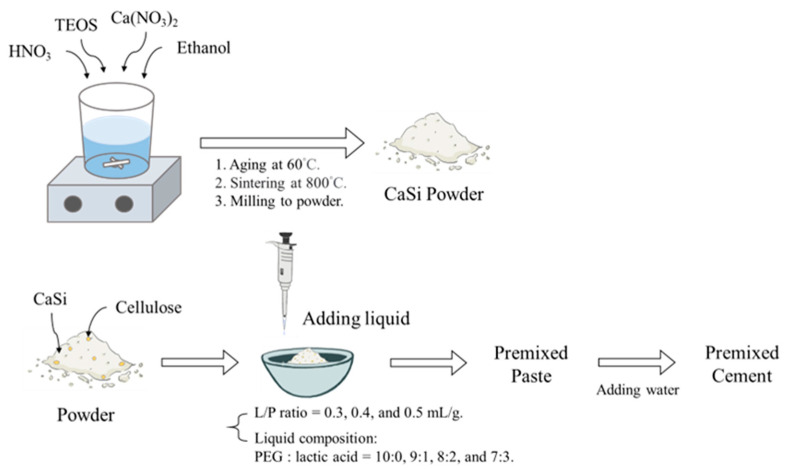
A schematic diagram of the experimental process.

Different formulations of premixed cement samples were designed with variable L/P ratios and lactic acid concentrations in polyethylene glycol 400 (PEG400). The low-molecular-weight grade PEG400 (Thermo, Heysham, UK) and lactic acid (MERCK, Darmstadt, Germany) were used. The liquid phase was composed of PEG400 and lactic acid in various PEG400/lactic acid volume ratios of 10:0, 9:1, 8:2, and 7:3. Simultaneously, the L/P ratio of paste was 0.3, 0.4, or 0.5 mL/g. Table 1 provides detailed information about the sample formulations, and the sample codes were created based on the L/P ratio and lactic acid (LA) amount. For example, the 4L20 sample had an L/P ratio of 0.4 mL/g and a lactic acid concentration of 20%. After thoroughly mixing the powder and water-free liquid, the mixture became a premixed paste. To imitate the scenario where the paste absorbs body fluids in the human body, we added a small amount of distilled water directly to the paste at a water–powder ratio of 0.147 mL/g [4]. Subsequently, the cement was placed in a 37 °C incubator with 100% humidity for one day to harden further. In short, Figure 1 illustrates the preparation process of CaSi powder, premixed paste with different component ratios, and cement.

### 2.3. Phase Composition and Surface Morphology 

The cement and paste samples were compressed into disks of 10 mm diameter. The phase composition was analyzed by X-ray diffractometry (XRD; Bruker D8 SSS, Karlsruhe, Germany) with Ni-filtered Cukα radiation. Fourier transform infrared spectroscopy (FTIR) was also carried out by blending the samples with KBr and compressing them into disks. The FTIR analysis was conducted using a Bruker Vertex 80v instrument (Bruker, Ettlingen, Germany) in the transmittance mode within the wavenumber range of 400 to 4000 cm^−1^. For surface morphology examination, the samples were coated with gold by a JFC-1600 coater (JEOL, Tokyo, Japan) and then observed with a field-emission scanning electron microscope (FESEM; JEOL JSM-7800F, Tokyo, Japan).

### 2.4. Setting Time

After mixing the paste with a small amount of distilled water, we transferred it into a Teflon mold with a 6-mm depth. A 400-g Gillmore needle with a 1 mm diameter was used to determine the setting time of the premixed cement. The setting time was recorded when water was added to the paste until the needle no longer made a perceptible indentation. Each sample underwent this test five times. 

### 2.5. Diametral Tensile Strength

The premixed cement samples were molded into disks with a diameter of 6 mm and a thickness of 3 mm. The diametral tensile strength (DTS) was tested by the EZ-Test machine (Shimadzu, Kyoto, Japan) at a loading rate of 0.5 mm/min. The strength value of each sample was calculated by the formula of S = 2P/πbw, where S represents strength (MPa), P is the peak load (N), b is the diameter of the sample (mm), and w is the thickness of the sample (mm). The peak load was gained from the recorded load–deflection curves. Five experiments were repeated. 

### 2.6. pH Value

To determine the pH value, a small amount of distilled water was added to the paste, and the pH meter (model SP-701, SUNTEX, New Taipei, Taiwan) was immediately placed in the cement. The pH value of each sample was then recorded. Three experiments were repeated.

### 2.7. Anti-Washout Ability of Paste

Premixed paste samples were molded using a cylindrical Teflon mold with a 6 mm diameter and 6 mm height and then compressed under 0.7 MPa uniaxial pressure for 1 min. After removal from the Teflon mold, each sample was immersed in 40 mL of distilled water. The anti-washout ability of each sample was determined through visual inspection. If the paste maintained its integrity after being immersed in water for 1 h, it was thought to have anti-washout ability.

### 2.8. Cytotoxicity 

Premixed cement samples and CaSi cement were ground into powders, sterilized by UV light overnight, and placed in transwell inserts (JET BIOFIL, Guangzhou, China). During cell culture, the transwell inserts were positioned in a 24-well plate and soaked in Dulbecco’s Modified Eagle Medium (DMEM; HyClone, Logan, UT, USA) containing 10% fetal bovine serum (FBS; HyClone) and 1% penicillin/streptomycin solution (Gibco, Langley, OK, USA). Negative and positive controls were established by placing wells with only DMEM and DMEM containing 10% dimethyl sulfoxide (DMSO), respectively. For this experiment, L929 was selected to test the cytotoxicity of the samples. We added 10,000 cells to each well and cultured them in DMEM for 12, 24, and 48 h in 5% CO_2_ at 37 °C. At the end of the culture period, the MTT (3-(4,5-dimethylthiazol-2-yl)-2,5-diphenyltetrazolium bromide; Sigma-Aldrich, St. Louis, MO, USA) assay was used to examine cell vitality by reading the optical density (OD) at a wavelength of 563 nm. The viability (%) was calculated using the following formula: Viability (%) = (OD of the sample)/(OD of negative control) × 100%. Three repeated experiments were performed to ensure the accuracy of the results. 

### 2.9. Bacteriostasis 

Premixed cement samples, CaSi cement, and calcium hydroxide were compressed into the bottom of a 48-well plate for sample preparation. One of the wells was left empty as the negative control, while calcium hydroxide served as the positive control. To ensure sterilization, the samples were exposed to UV light for 1 h before adding a bacterial suspension. Bacteriostatic tests were conducted using *S. aureus* and *E. coli*, which were cultured in tryptic soy broth (Beckton Dickinson, Sparks, MD, USA) at 37 °C, and then diluted to a concentration of 10^6^ CFU/mL. Each sample was cultured with 1 mL of bacterial suspension for 3 and 24 h. At the end of culturing, the bacterial suspension medium of each sample was tested by Alarmar blue assay (Invitrogen, Grand Island, NY, USA) and examined for OD at a wavelength of 570 nm with a reference wavelength of 600 nm. The bacteriostatic ratios of samples were gained from the following formula: Bacteriostatic ratio (%) = (OD of negative control − OD of the sample)/OD of negative control × 100%. Additionally, the pH values of the culture medium were tested by a pH meter (LAQUAtwin; HORIBA, Tokyo, Japan) after 3 and 24 h of culturing with the samples.

### 2.10. Statistical Analysis

The results of all the experiments were presented as mean values with standard deviation. One-way ANOVA and Scheffe’s multiple comparison tests were used to compare the differences between the means of experimental and control groups. If the *p*-value was less than 0.05, we considered it a significant difference.

## 3. Results

### 3.1. Phase Composition 

**Figure 2:**
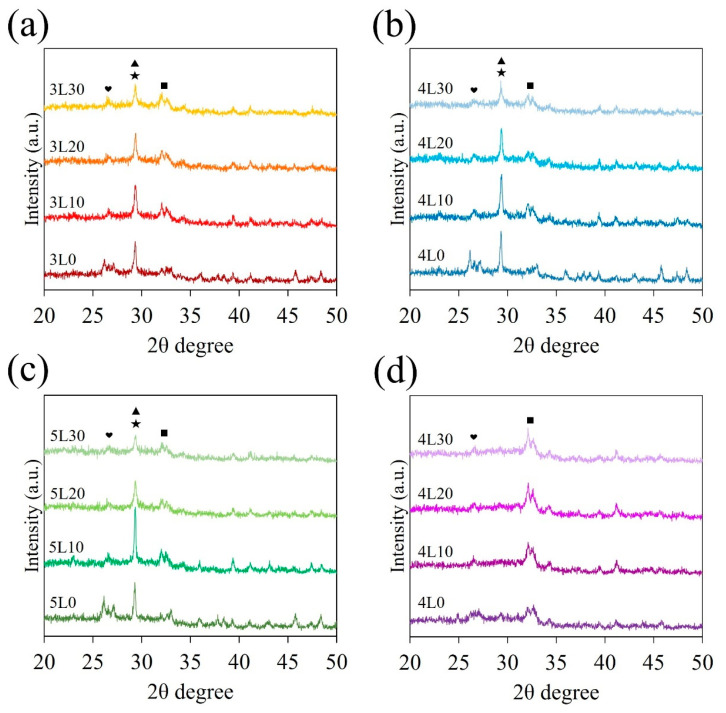
XRD patterns for (**a**) 3L, (**b**) 4L, and (**c**) 5L cement samples, as well as (**d**) 4L paste samples. (symbols: ■: β-Ca_2_SiO_4_, ❤: CaSiO_3_, ★: C-S-H, and ▲: CaCO_3_).

The XRD patterns of all the premixed cement samples are exhibited in Figure 2a–c. A sharp peak at 2θ = 29.4° was observed in all the premixed cement samples, corresponding to the overlapping peaks of calcium silicate hydrate (C-S-H) and CaCO_3_ [31]. However, this peak was weaker in samples 3L30, 4L30, and 5L30. The broad peak in the range of 2θ = 32°–34° represented the incompletely reacted inorganic component phase of β-Ca_2_SiO_4_ [32], while the peak at 2θ = 26.6° corresponded to CaSiO_3_ [31]. Additionally, Figure 2d shows that the 4L paste samples only exhibited the peaks of β-Ca_2_SiO_4_ and CaSiO_3_ because they did not undergo a hydration reaction, unlike the 4L cement samples shown in Figure 2b. 

**Figure 3:**
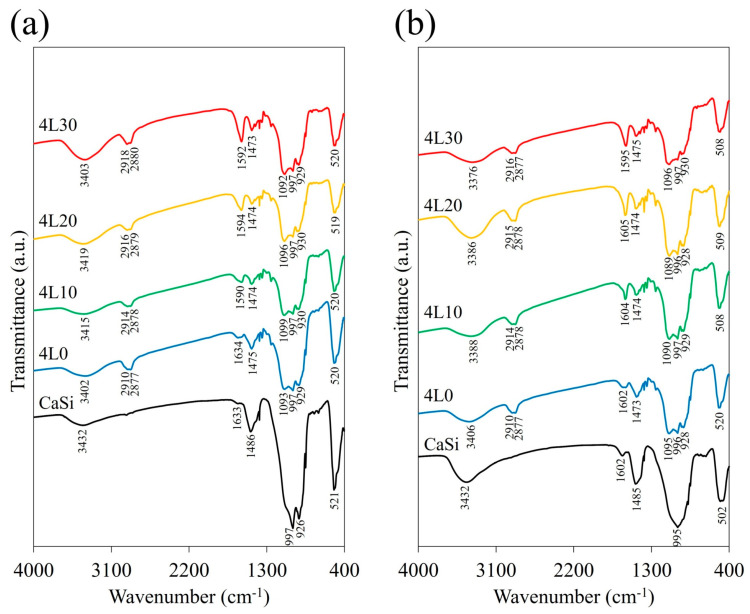
FTIR spectra of (**a**) 4L paste samples and CaSi powder and (**b**) 4L cement samples and CaSi cement.

The FTIR spectra of the premixed paste samples of the 4L group (4L0, 4L10, 4L20, and 4L30) and CaSi powder are shown in Figure 3a, while the corresponding spectra of the cement samples are exhibited in Figure 3b. The CaSi powder had an absorption peak at 521 cm^−1^ ascribed to the bending or rocking of Si–O–Si [33]. Moreover, the broad and intense band ranging from 900 to 1100 cm^−1^ corresponded to the stretching vibration of Si–O–Si [33], overlapping with the peak of Si–OH in 900 to 980 cm^–1^ [34]. The peak of CO_3_ in 1475 cm^−1^ might come from the atmospheric carbonation of CaSi [35]. CaSi cement, compared to CaSi powder, had a more prominent peak at 3400 cm^−1^, which was attributed to a hydroxyl group in H_2_O (Figure 3b). It is reasonable that the peaks/bands of CaSi also appeared in the FTIR spectra of the premixed paste and cement but were less intense. Nevertheless, premixed paste and cement samples had other absorption bands, such as 2900 cm^−1^, which were related to the alkyl groups of CH_2_ and CH_3_ from PEG400, lactic acid, and cellulose [36,37]. In addition, they also had a more prominent peak around 1600 cm^−1^ related to the stretching vibration of the carboxyl group (COO^–^) from lactic acid and cellulose [38]. The intensity of this peak increased with the higher lactic acid concentrations of the samples. The peak of the hydroxyl group appeared in the premixed paste and cement samples, coming from not only H_2_O but also cellulose and lactic acid [19,36,38].

### 3.2. Surface Morphology

**Figure 4:**
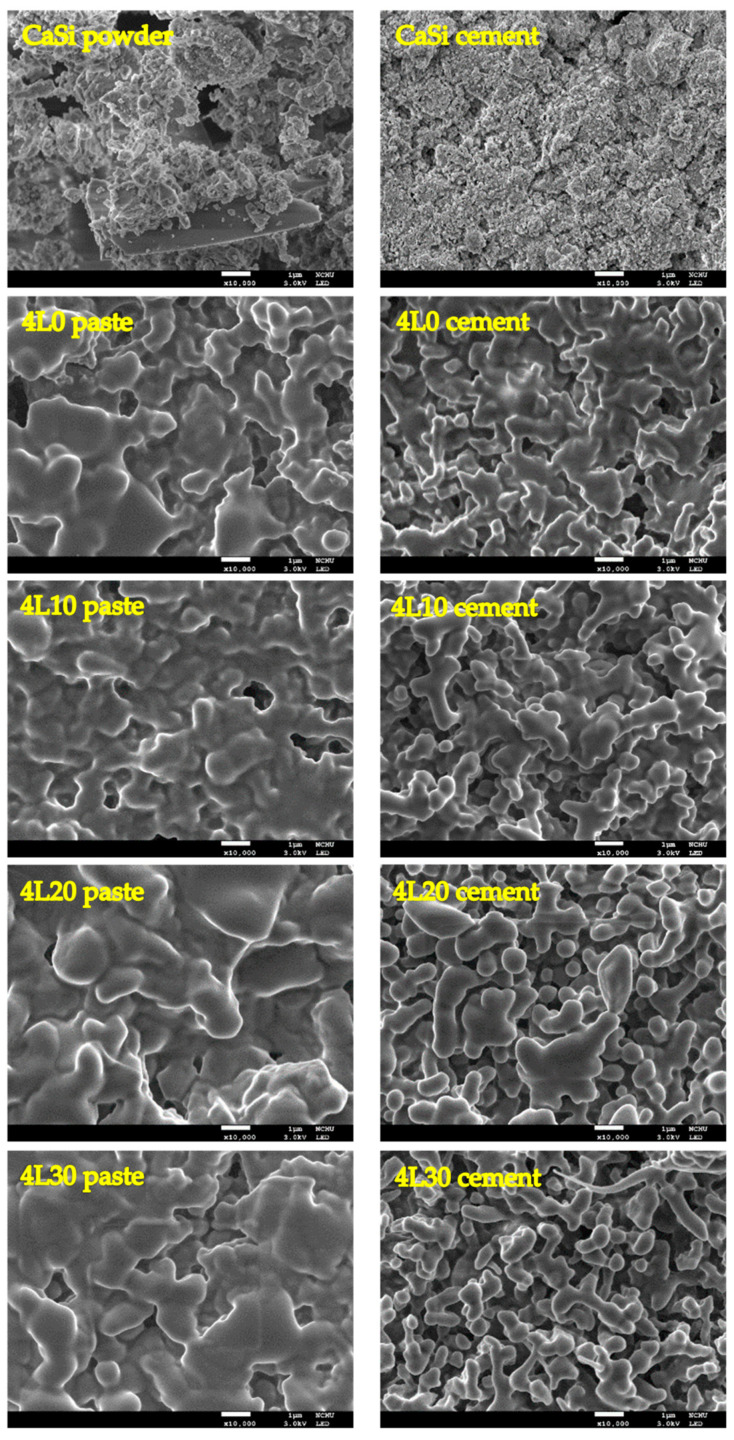
FESEM micrographs of CaSi powder and 4L premixed paste with corresponding cement types. Scale bar: 1 μm.

The FESEM images of CaSi and the 4L group before and after setting are shown in Figure 4. Compared to CaSi powder with irregular particles, CaSi cement exhibited the morphology of aggregated particles due to the setting reaction. On the other hand, the premixed paste and cement samples of the 4L group displayed smooth and liquid-like surfaces. However, there was a noticeable difference between premixed paste and cement type. The premixed cement samples had smaller clusters with porous structures. Furthermore, 4L30 premixed cement appeared to have even smaller clusters than the other premixed cement samples.

### 3.3. Setting Time

As outlined in Table 1, the setting times of 4L10, 4L20, and 4L30 are 94, 11.2, and 2.8 min, respectively. These resulted indicated that samples with higher lactic acid concentrations tended to have shorter setting times. The 3L and 5L groups followed a similar setting time trend as those of the 4L group. However, it is worth noting that only in the 4L and 5L groups showed a significant (*p* < 0.05) decrease in setting time with increasing lactic acid concentrations. Regarding the L/P effect, 3L20, 4L20, and 5L20 samples displayed a significantly (*p* < 0.05) increased setting time with higher L/P ratios. Out of all the samples, 3L0, 3L10, 3L20, 3L30, 4L20, 4L30 and 5L30 showed setting times shorter than 15 min. Conversely, the remaining groups had setting times much longer than 60 min. 

### 3.4. Diametral Tensile Strength

The diametral tensile strength (DTS) of all the premixed cement samples is presented in Table 1. Notably, there was a consistent increase in the DTS of the samples with the rise in lactic acid concentrations, regardless of the L/P ratios. This difference was found to be significant (*p* < 0.05) across the samples. For instance, the strength values for 4L0, 4L10, 4L20, and 4L30 were 0.02, 0,08, 0.2, and 0.4 MPa, respectively. Furthermore, it was observed that the L/P ratios appeared to have no discernible impact on the DTS values. 3L30, 4L30, and 5L30 had DTS values ranging from 0.37 to 0.4 MPa, for which there was no significant difference (*p* < 0.05). 

### 3.5. pH Value

The pH values of all the samples are also exhibited in Table 1. A decline in pH value was observed as the lactic acid concentration increased. In the 4L group, the pH values of 4L0, 4L10, 4L20, and 4L30 were recorded as 10.5, 9.8, 9.6, and 9.2, respectively, and this difference was found to be statistically significant (*p* < 0.05). The pH values of the 3L and 5L groups followed a similar trend as the 4L group. Additionally, the L/P ratio had a negligible influence on pH level.

### 3.6. Anti-Washout Ability

**Figure 5:**
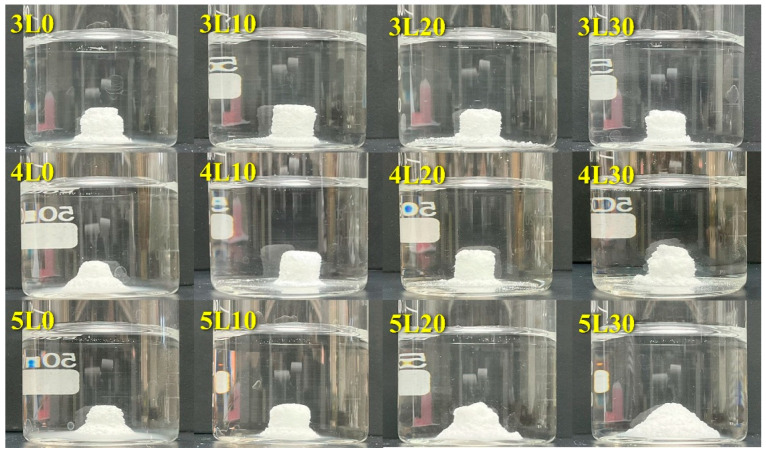
Anti-washout properties of all premixed paste samples.

In Figure 5, we assessed the anti-washout ability of each sample after one hour of immersion in distilled water. If the cement retained its initial shape, it was deemed to have anti-washout properties. The 5L group showed no anti-washout properties, indicating that a higher L/P ratio weakened the washout resistance of the premixed cement. Similarly, the samples without lactic acid (3L0, 4L0, and 5L0) disintegrated, although they contained the gelling agent cellulose. Thus, lactic acid may play an important role in anti-washout properties. However, sample 4L30 demonstrated inadequate washout resistance, implying that excessive lactic acid may compromise its anti-washout ability. This underscored the importance of maintaining an appropriate lactic acid concentration in the premixed cement to resist washout behavior. In summary, samples 3L10, 3L20, 3L30, 4L10, and 4L20 exhibited acceptable anti-washout ability. Taking the anti-washout property and setting time into consideration, we chose 3L10, 3L20, 3L30, and 4L20 for further in vitro studies of the cytotoxicity and bacterial inhibition. Sample 4L10 was excluded due to its notably longer setting time (94 min). 

### 3.7. Cytotoxicity

**Figure 6:**
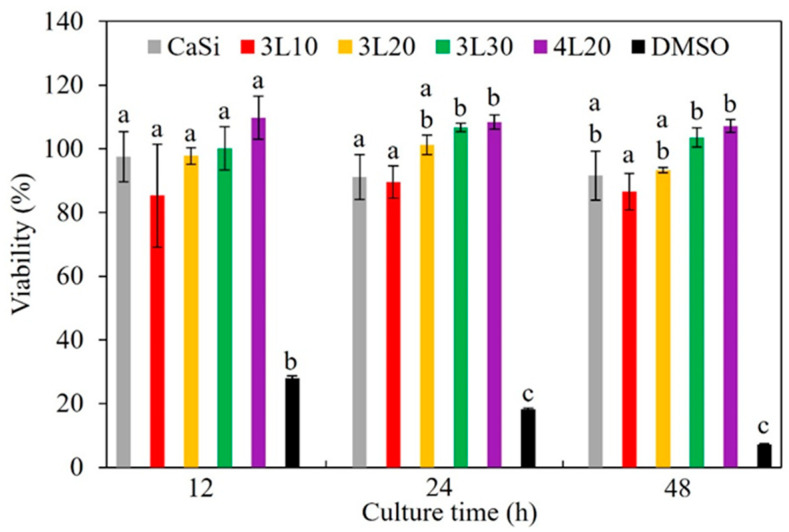
Viability of L929 cells cultured in DMEM with various premixed cement samples for 12, 24, and 48 h. Statistical comparisons were made between samples at the same culture time. Different letters above the bars in this figure represent a significant difference (*p* < 0.05).

The viability (%) in Figure 6 is calculated based on the absorbance values of the samples, which can reflect the number of viable cells. Lower viability indicates higher cytotoxicity of the sample. The 10% DMSO was used as a positive control and exhibited a meager viability of 28% to 7% across the three culture time points. In contrast, all the cement samples exhibited viability exceeding 80%, suggesting minimal cytotoxicity and high biocompatibility. Between the culture times of 12 and 48 h, 4L20 demonstrated the highest viability at 107–110%, whereas 3L10 exhibited the lowest viability at 85% to 90% among all the samples. However, these differences were not statistically significant (*p* > 0.05).

### 3.8. Anti-Bacterial Property 

**Figure 7:**
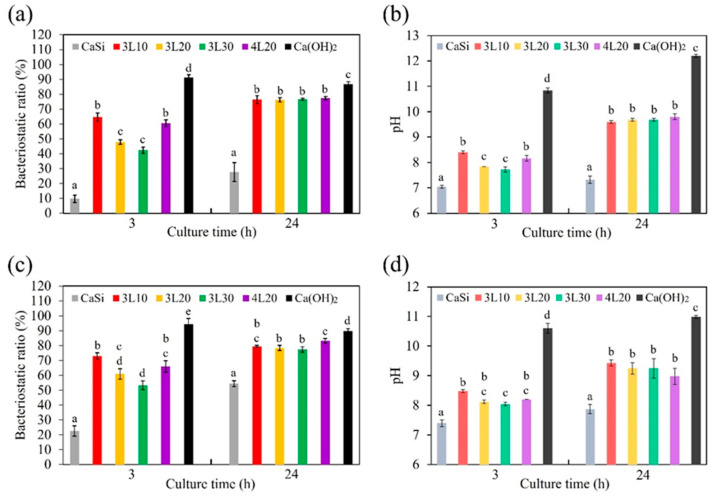
Bacteriostatic ratio (%) of premixed cement samples against (**a**) *S. aureus* and (**c**) *E. coli* after culturing for 3 and 24 h, as well as corresponding changes (**b,d**) in pH values of culture broth. Statistical comparisons were made between samples at the same culture time. Different letters above the bars in the figures represent a significant difference (*p* < 0.05).

Figure 7 illustrates the bacteriostatic ratios (%) of the samples and corresponding pH values of the culture medium when tested against *S. aureus* and *E. coli* after 3 and 24 h of culture. The bacteriostatic ratios were calculated based on the absorbance values of the samples. Calcium hydroxide had the highest bacteriostatic ratio of 87% to 94% against both bacterial species at the two culture time points, being a positive control. In contrast, premixed cement samples had significantly higher (*p* < 0.05) bacteriostatic ratios than CaSi cement. When testing against *S. aureus* (Figure 7a), lower lactic acid concentrations led to improved antibacterial efficacy for short-term culture. Specifically, after 3 h of culture, the bacteriostatic ratios of 3L10, 3L20, and 3L30 were 65%, 48%, and 42%, respectively. Notably, the bacteriostatic ratio of 4L20 was close to that of 3L10. However, after 24 h of culture, all the premixed samples displayed a consistent bacteriostatic ratio of approximately 76%. Interestingly, the pH values followed a trend similar to the bacteriostatic ratios across the two culture time points (Figure 7b). As an example, after 3 h of culture, the pH values of 3L10 and 4L20 were 8.4 and 8.2, respectively, significantly higher (*p* < 0.05) than those of 3L20 (pH 7.8) and 3L30 (pH 7.7). After 24 h, the pH values of premixed cement samples remained steady at approximately 9.7, higher than those at 3 h. Compared to premixed cement samples, Ca(OH)_2_ made the culture medium highly alkaline, while the culture medium with the CaSi sample exhibited the least alkalinity. The bacteriostatic tests and pH values of the culture medium against *E. coli* (Figure 7c,d) showed a similar trend to those against *S. aureus*.

## 4. Discussion

In clinical practice, preparing conventional bone cement by mixing water and powder often makes procedures complicated. However, premixed cement has been invented to simplify the use of bone cement during surgery. Studies on premixed CaSi cement are ongoing in the rapidly advancing world [24,25,39,40]. Nevertheless, specific challenges have persisted in previous research papers. For instance, premixed CaSi bone cement typically requires a setting accelerator for faster hardening [24]. Additionally, the washout resistance of CaSi remains suboptimal, making it susceptible to being washed away by body fluids [2]. Aside from these handling properties, the antibacterial ability of CaSi cement also needs improvement. Hence, it is essential to research and develop a new type of premixed CaSi cement. Simultaneously, the evaluation of its cytotoxicity was crucial to ensure its biocompatibility. Furthermore, an in-depth examination of the physicochemical characteristics was essential to a better understanding of the interactions between the different materials within the premixed CaSi cement. 

Understanding the phase composition is essential for comprehending the chemical reactions in premixed cement. Analysis of the XRD patterns revealed that β-Ca_2_SiO_4_ consistently showed a higher peak than CaSiO_3_, indicating that the primary component of CaSi from the sol–gel method was dicalcium silicate. The differences between phases in premixed cement and paste were significant due to hydration reactions. When mixed with water, CaSi formed C-S-H and Ca(OH)_2_. Subsequently, Ca(OH)_2_ underwent carbonation in the air and transformed into CaCO_3_. However, the peak in samples 3L30, 4L30, and 5L30 weakened due to the dissolution of C-S-H and CaCO_3_ by acid solutions [41]. 

The premixed cement contained various ingredients, such as cellulose, PEG400, and lactic acid, which were in either polymer or liquid form. Analyzing these components via XRD has been challenging for scrutinizing differences. Hence, FTIR was performed to explore these components in premixed cement better. Compared to CaSi, the premixed paste and cement exhibited additional peaks of alkyl groups and carboxyl groups due to cellulose, lactic acid, and PEG400 in the premixed paste and cement. Furthermore, in samples with increasing lactic acid concentrations, the peak at 1600 cm^−1^ became sharper, representing the carboxyl groups of lactic acid. Additionally, the broad peak representing the hydroxyl group was higher in CaSi cement than in CaSi powder due to the hydration reaction. However, this peak in the FTIR spectra of the paste and premixed cement was similar, possibly due to the absorption of moisture from the air by the premixed paste samples. Apart from the phase composition, the surface morphology was also important. Premixed paste and cement had a liquid-like and smooth surface compared to CaSi cement. This difference arose from including PEG400 as a polymer liquid in the premixed samples. In addition, after mixing the premixed paste with water, the surface of the cement looked more porous, and the clusters, referring to the agglomerations of cement particles, became smaller. This occurrence was linked to the dissolution effect following the interaction of cement with water and lactic acid. More obviously, 4L30 displayed even smaller clusters when compared to other premixed cement samples due to the pronounced dissolution effect from the acidity of 30% lactic acid [41].

Setting time is an essential characteristic of cement in clinical applications as it affects the duration of patients’ visits to clinics. It would be beneficial if premixed cement could harden rapidly when filling bone defects. Typically, a setting accelerator is required for premixed CaSi cement. In this study, lactic acid was selected as a setting accelerator. It not only reduced the setting time and pH but also improved the strength of the premixed cement. Previous research has shown that the setting time of CaSi cement is around 20 min [31], which is longer than the required 10 min in clinical settings [2,42]. In this study, the premixed cement samples with lower L/P ratios and higher lactic acid concentrations exhibited significantly shorter setting times. Specifically, the 3L group, 4L20, 4L30, and 5L30 had setting times shorter than that of CaSi, making these premixed cement samples more suitable for clinical use. Unsurprisingly, the smaller L/P ratio contributed to a shorter setting time, consistent with a previous study [43]. Also, when lactic acid reacted with CaSi, a precipitation reaction may have occurred with the formation of calcium lactate to facilitate the hardening of CaSi [26,27]. 

The diametral tensile strength test is an indirect tensile test designed to reflect the compressive and tensile properties of a material. It involves applying a compressive force in the load direction across the specimen diameter, thereby introducing tensile stress along its central diameter [35,44,45]. The strength value of CaSi cement was 2.5 MPa [31], while premixed cement samples exhibited strengths below 0.5 MPa. The lower value in premixed cement could be attributed to its elevated polymer content, which may result in inherent weakness. Furthermore, an interesting finding is that a higher lactic acid concentration in premixed cement samples resulted in greater strength, but the L/P ratio had minimal impact on the strength. However, the lactic acid concentration in the cement sample presented an inverse correlation with the pH value. This is because lactic acid neutralized the alkaline properties of CaSi, which has a pH value of 11.7 [10] and releases calcium hydroxide during the hydration reaction. 

The ability of cement to resist being washed out is crucial in medical applications. This property ensures that the cement remains stable when exposed to body fluids, making it easier for clinicians to work with [2]. Our research discovered that an increased L/P ratio of the cement samples decreased their ability to resist washout. This could be because the premixed cement with a higher L/P ratio became more porous, making it more susceptible to fluid penetration and structure breakdown. On the other hand, appropriate concentrations of lactic acid were beneficial for the washout resistance of the cement samples. Specifically, 3L10, 3L20, 3L30, 4L10, and 4L20 showed promising results, possibly due to the precipitation product. However, an excessive lactic acid concentration may cause the premixed cement to dissolve or become etched, leading to increased porosity, as demonstrated in the FESEM image of 4L30. Therefore, premixed cement with an appropriate lactic acid concentration could exhibit the best washout resistance.

Apart from handling properties like setting time and washout characteristics, it is essential to consider the material’s cytotoxicity for clinical application. When the material is implanted, its chemicals are gradually released into the surrounding tissue, affecting the cell vitality. ISO 10993-5 recommends a non-cytotoxic viability value above 70%. The tested samples, including 3L10, 3L20, 3L30, and 4L20, exhibited more than 80% viability, as documented in the evaluation of L929 cells. Furthermore, there was almost no significant difference between the viability values of all tested samples during the culture time points. It is well recognized that the CaSi control is proven to be an osteogenic ceramic both in vitro and in vivo [19,20,46,47,48]. Lactic acid aids in reducing free radicals and improving cell proliferation [49,50], and FDA-approved PEG is non-toxic. It is, therefore, no surprise that all the tested premixed samples demonstrated high biocompatibility.

**Figure 8:**
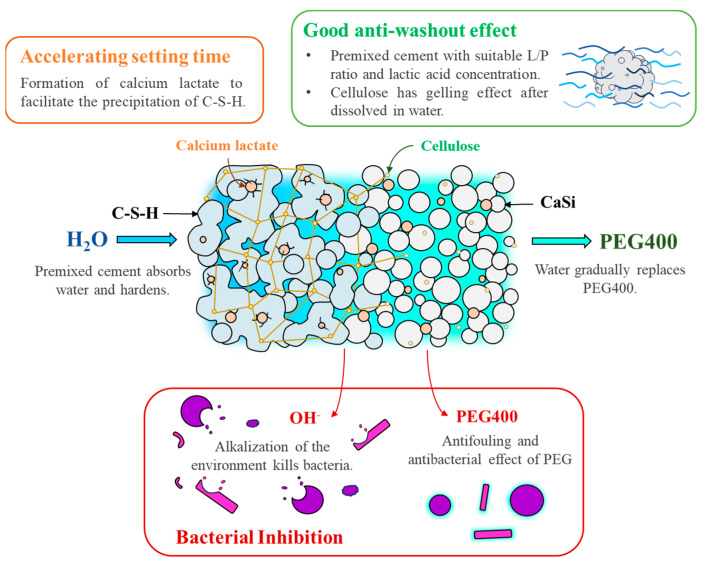
The schematic diagram illustrating the multiple roles of PEG and lactic acid in the premixed CaSi cement.

The ability of bone cement to fight bacteria is crucial for reducing the risk of infection after surgery. Both Gram-positive and Gram-negative bacteria, *S. aureus* and *E. coli*, respectively, were selected for the experiments. We assessed the short-term (3 h) and long-term (24 h) antibacterial effectiveness of premixed cement samples. Our findings showed that calcium hydroxide exhibited the best antibacterial ability due to its high pH value. All premixed cement samples demonstrated superior antibacterial ability compared to the CaSi control. This superiority was attributed to releasing more alkali into the culture broth by the premixed cement samples, as confirmed by the broth’s pH value. The increased alkali release likely resulted from the gradual fluid exchange between the entire premixed cement and the culture broth. In contrast, CaSi cement can only release alkali from the exposed surface. After 3 h of culture, samples with lower lactic acid concentration showed better antibacterial ability as lactic acid could lower the pH value, reducing its antibacterial ability. Additionally, previous studies have shown that PEG has antibacterial ability [51,52]. When seeding for 24 h, the bacteriostatic ratios of all premixed cement samples and the pH of the broth significantly increased and became consistent. This could be because the alkali of the premixed cement continued to be released into the broth over a long period. Another factor is the pH homeostasis in metabolism during a long culture period, regardless of the initial broth pH [53,54]. In summary, variations in the pH of broth containing premixed cement only happened for a short culture period. A comprehensive diagram illustrating the various roles of PEG and lactic acid in enhancing the properties of premixed CaSi cement is depicted in Figure 8. 

## 5. Conclusions

Premixed cement has been developed to facilitate clinical operations. However, the handling properties and antibacterial efficacy of such bone cement are still unmet clinical needs. This study used a combination of PEG and lactic acid as a liquid phase to synergistically improve the two crucial properties of premixed CaSi cement. Suitable concentrations of lactic acid effectively reduced the setting time and enhanced the washout resistance. Within the limit of this study, the premixed cement exhibited non-cytotoxicity. Regarding bacteriostasis, the presence of polyethylene glycol and lactic acid greatly increased the antibacterial ability of the CaSi cement. Based on its setting time, washout resistance, and antibacterial activity, the 4L20 premixed cement with a liquid phase of 80% PEG and 20% lactic acid at an L/P ratio of 0.4 mL/g holds promise for dental and orthopedic practices, as it can potentially improve the efficiency and effectiveness of current bone cement. Further research will focus on the in vivo responses of premixed cement before clinical use.

## Figures and Tables

**Table 1:** Formulations of premixed cement samples with their corresponding setting time, diametral tensile strength, and pH value.

Sample Code	L/P	Liquid		Setting Time (min)	DTS (MPa)	pH Value
		PEG (vol%)	LA (vol%)			
**3L0** **3L10** **3L20** **3L30**	0.3	100	0	6.6 ± 0.9 ^a,b^	0.04 ± 0.00 ^a,b^	10.3 ± 0.2 ^a,b^
		90	10	5.4 ± 0.5 ^a,b^	0.10 ± 0.02 ^b^	9.7 ± 0.3 ^a,b,c^
		80	20	2.8 ± 0.4 ^b^	0.26 ± 0.04 ^c^	9.6 ± 0.2 ^a,b,c^
		70	30	1.8 ± 1.1 ^b^	0.37 ± 0.05 ^d^	9.4 ± 0.3 ^b,c^
**4L0** **4L10** **4L20** **4L30**	0.4	100	0	N/A	0.02 ± 0.00 ^a^	10.5 ± 0.5 ^a^
		90	10	94.0 ± 5.5 ^c^	0.08 ± 0.02 ^a,b^	9.8 ± 0.3 ^a,b,c^
		80	20	11.2 ± 3.0 ^a,d^	0.20 ± 0.02 ^c^	9.6 ± 0.1 ^a,b,c^
		70	30	2.8 ± 0.4 ^b^	0.40 ± 0.03 ^d^	9.2 ± 0.3 ^c^
**5L0** **5L10** **5L20** **5L30**	0.5	100	0	N/A	0.01 ± 0.00 ^a^	10.4 ± 0.1 ^a,b,^
		90	10	N/A	0.08 ± 0.01 ^a,b^	9.5 ± 0.1 ^a,b,c^
		80	20	94.0 ± 5.5 ^c^	0.19 ± 0.01 ^c^	9.4 ± 0.2 ^b,c^
		70	30	14.4 ± 0.9 ^d^	0.39 ± 0.05 ^d^	9.1 ± 0.4 ^c^

N/A means “no setting after 2 h”. Values are means ± standard deviation. The same superscript letter (a, b, c, and d) in a column following values represents no significant difference (*p* > 0.05) between them according to Scheffe’s multiple comparisons.

## Data Availability

The original contributions presented in the study are included in the article, further inquiries can be directed to the corresponding authors.
